# A Systematic Review and Meta-analysis of the Therapeutic Effect of Acupuncture on Migraine

**DOI:** 10.3389/fneur.2020.00596

**Published:** 2020-06-30

**Authors:** Ming-Qian Ou, Wei-Hao Fan, Fu-Rong Sun, Wan-Xin Jie, Mei-Jun Lin, Yu-Jie Cai, Shi-Yun Liang, Yang-Sheng Yu, Min-Hua Li, Li-Li Cui, Hai-Hong Zhou

**Affiliations:** ^1^Department of Neurology, Affiliated Hospital of Guangdong Medical University, Zhanjiang, China; ^2^Department of Neurology, Luoding People's Hospital, Affiliated Hospital of Guangdong Medical University, Luoding, China; ^3^Guangdong Key Laboratory of Age-Related Cardiac and Cerebral Diseases, Affiliated Hospital of Guangdong Medical University, Zhanjiang, China; ^4^Department of Neurology, Puning People's Hospital, Southern Medical University, Jieyang, China

**Keywords:** acupuncture, migraine, efficacy and safety, transcranial doppler, systematic review, meta-analysis

## Abstract

**Background:** Migraine is an intractable headache disorder, manifesting as periodic attacks. It is highly burdensome for patients and society. Acupuncture treatment can be beneficial as a supplementary and preventive therapy for migraine.

**Objectives:** This systematic review and meta-analysis aimed to investigate the efficacy and safety of acupuncture for migraine, and to examine transcranial doppler changes after acupuncture.

**Methods:** Reports, conference, and academic papers published before March 15, 2019 in databases including PubMed, Cochrane library, Embase, China National Knowledge Infrastructure, WANFANG Database, Chinese journal of Science and Technology, and China Biomedical were searched. Randomized controlled trials (RCTs) involving acupuncture, sham acupuncture, and medication in migraine were included. The Cochrane Collaboration software, RevMan 5.3, was used for data processing and migration risk analysis.

**Results:** Twenty-eight RCTs were included. 15 RCTs included medication only, 10 RCTs included sham acupuncture only, and 3 RCTs included both. The study included 2874 patients, split into 3 groups: acupuncture treatment group (*n* = 1396), medication control group (*n* = 865), and sham acupuncture control group (*n* = 613). The results showed that treatment was more effective in the acupuncture group than in the sham acupuncture group (MD = 1.88, 95% CI [1.61, 2.20], *P* < 0.00001) and medication group (MD = 1.16, 95% CI [1.12, 1.21], *P* < 0.00001). Improvement in visual analog scale (VAS) score was greater in the acupuncture group than in the sham acupuncture group (MD = −1.00, 95% CI [-1.27,−0.46], *P* < 0.00001; MD = −0.59, 95% CI [-0.81,−0.38], *P* < 0.00001), and their adverse reaction rate was lower than that of the medication group (RR = 0.16, 95% CI [0.05, 0.52], *P* = 0.002). The improvement of intracranial blood flow velocity by acupuncture is better than that by medication, but the heterogeneity makes the result unreliable.

**Conclusions:** Acupuncture reduced the frequency of migraine attacks, lowered VAS scores, and increased therapeutic efficiency compared with sham acupuncture. Compared with medication, acupuncture showed higher effectiveness with less adverse reactions and improved intracranial blood circulation. However, owing to inter-study heterogeneity, a prospective, multicenter RCT with a large sample is required to verify these results.

## Introduction

Migraine is characterized by recurrent, pulsating headaches and elevated intracranial blood flow caused by vasomotor and cerebrocortical dysfunction, vasospasm, and excessive stress. It can be triggered by stimuli such as light, sound, or physical activity (1). Symptoms such as nausea and vomiting can occur with the aggravation of the condition. In severe cases, patients may also develop neuropsychiatric symptoms (2). Migraine pathophysiology mainly involves functional changes occurring within the trigeminal neurovascular system, which includes the trigeminal ganglion, the meningeal vascular system, and specific nuclei of the brain stem, thalamus, and somatosensory cortex. The currently available pharmacological treatments for acute migraine are mainly aimed at controlling blood vessel dilation, trigeminal nerve activation, and serotonin (5-hydroxytryptamine) signaling (3–5). However, these drugs seem to be unable to fully counteract the complex pathological mechanisms underlying the condition, making recurrence possible. Further, these treatments are associated to adverse effects that may negatively affect patient prognosis. In addition to these challenges, migraine treatment is associated with high medical costs and resource use (6). In the Global Burden of Disease Study 2016, migraine was ranked as the sixth most disabling disease (7), indicating its huge adverse impact on individuals and society.

Presently, the acute stage of migraine is principally treated with non-steroidal anti-inflammatory drugs (NSAIDs), barbiturates, and opioids. Although the above drugs have achieved positive results in the treatment and prevention of migraine, some side effects are inevitable for patients with long-term use. The medication overuse headache (MOH) will be generated after long term use of NSAIDs. Additionally, the ergotamine may cause nausea, vomiting, vertigo, restlessness, gastric, and chest symptoms, whereas an overdose or chronic overuse may precipitate MOH, ergotism as well as heart and brain infarction (8, 9). In view of the fact that migraine may be chronic, thus requiring long-term control, it is relevant for current research to explore treatment scheme focusing on high efficacy and minimal side effects. In Asian countries such as China, traditional Chinese therapies, especially acupuncture, herbal medicine, and massage, have been shown to confer unique advantages in the treatment of migraine (10–12). In Western countries, acupuncture has also gradually been accepted as a viable alternative or supplementary therapy for migraine and other pain conditions (13, 14). Prior research has shown that acupuncture can improve migraine and associated cutaneous allodynia by inhibiting the expression of calcitonin gene-related peptide in the trigeminal ganglion, and by alleviating neuroinflammation through the reduction of inflammatory factor levels such as those of interleukin-1 and tumor necrosis factor-α (15). Compared with medication, acupuncture therapy is relatively low-cost and has fewer side effects, making it an attractive option for the auxiliary regulation and prevention of various chronic diseases (16).

While acupuncture demonstrably attenuates and prevents migraine, and improves intracranial blood circulation, its curative effect in migraine remains controversial, due to the periodicity of the condition (17). In order to better understand the clinical effect of acupuncture on migraine and its influence on intracranial hemodynamics, we have performed a meta-analysis of randomized controlled trials (RCTs) of acupuncture treatment for migraine in the last ten years. Meta-analysis of 28 articles showed that acupuncture treatment has higher treatment efficiency than sham acupuncture treatment, and acupuncture can reduce the frequency of migraine attacks and ameliorate the visual analog scale (VAS) score more significantly. Compared with the medication (medication group), acupuncture treatment is more effective and the incidence of adverse reactions is lower. In addition, the transcranial doppler (TCD) analysis results suggested that the acupuncture group has a better hemodynamic improvement effect than the medication group, but the results need to be further verified due to the existence of heterogeneity.

## Methods

### Search Strategy

Literature databases such as PubMed, Cochrane Library, Embase, Web of Science, China National Knowledge Infrastructure (CNKI), WANFANG, Chinese Journal Of Science And Technology (VIP), and China Biomedical (CBM) were queried for RCTs of acupuncture treatments of migraine, and supplementary literature was manually retrieved. Search terms were limited to migraine and acupuncture. We included Chinese reports published between January 1, 2009–March 15, 2019. Due to the relative dearth of research on acupuncture treatment performed outside of China, we extended the acceptable time of publication of articles written in languages other than Chinese to January 1, 2000.

### Criteria for Selecting Articles

We employed the following inclusion criteria when selecting reports: (1) patients were diagnosed with “migraine” according to clear diagnostic (inclusion) criteria, regardless of age, gender, duration, and source of cases and did not have any other diseases; (2) the experimental group was treated with acupuncture (electro-acupuncture, ear acupuncture, puncture and bloodletting, or abdominal needle), while the control group was treated with sham acupuncture or drug therapy; (3) study was either an RCT or clinical controlled trial; and (4) patients' condition was evaluated by standardized efficacy evaluation criteria (attack frequency, duration, VAS score, TCD, efficacy rate, etc.). While reports written in any language were accepted, we employed the following exclusion criteria: (1) animal experiments, (2) repeated experiments, (3) no clear diagnostic criteria for migraine, (4) irregular evaluations of the patient's condition, and (4) the application of other treatments in addition to acupuncture (such as acupuncture combined with traditional Chinese medicine or moxibustion) in the treatment group.

### Data Extraction

The following data was obtained from the selected reports and inputted into an Excel template: frequency of migraine (FM), duration of migraine (DM), TCD, VAS, response rate (ER), and adverse reactions (AE). Both the selection of the reports and the extraction of the data were conducted independently by two different evaluators according to the inclusion and exclusion criteria; differences between the two evaluators were resolved through discussion, and the consistency of data was confirmed by a third party.

### Risk of Bias Assessment

Two researchers conducted quality evaluation of the included literature by using a risk-bias assessment tool provided by Cochrane Revman 5.3, and made judgments of high risk, low risk and unclear for each item. Bias types included (1) random sequence generation, (2) allocation hiding, (3) blinded researchers and subjects, (4) blinded comparison of the study results, (5) integrity of final data, (6) selective reporting of research results, (7) other sources. For example, in the generation of random sequences, high risk refers to the random sequence methods used by researchers to describe errors, such as classification by date of birth and outpatient number. Low risk refers to classification according to random number tables, computer random generators, coin flips, and so forth.

### Quality Assessment

Meta-analysis was performed using Cochrane systematic Review software Manage 5.3. Mean difference (MD) and 95% confidence intervals (CIs) were used to represent the classification variables. The risk ratio (OR) and its 95% CI were used to represent the counting data: OR= 1 indicated no difference between the two groups; OR > 1, high efficiency in the experimental group; and OR <1, high efficiency in the control group. The inter-study heterogeneity was tested with a chi-square test. *P* > 0.1 and I^2^ < 50% indicated that the inter-study heterogeneity was not statistically significant, in which case the fixed-effect model was adopted; otherwise, the random effect model was adopted. All effect quantities were expressed as 95% CIs, and a funnel plot was used to analyze whether publication bias existed in the literature.

### Ethical Statement

All studies were approved by our institution's ethics committee; no additional ethical statements are required.

## Results

### Search Results

**Figure 1:**
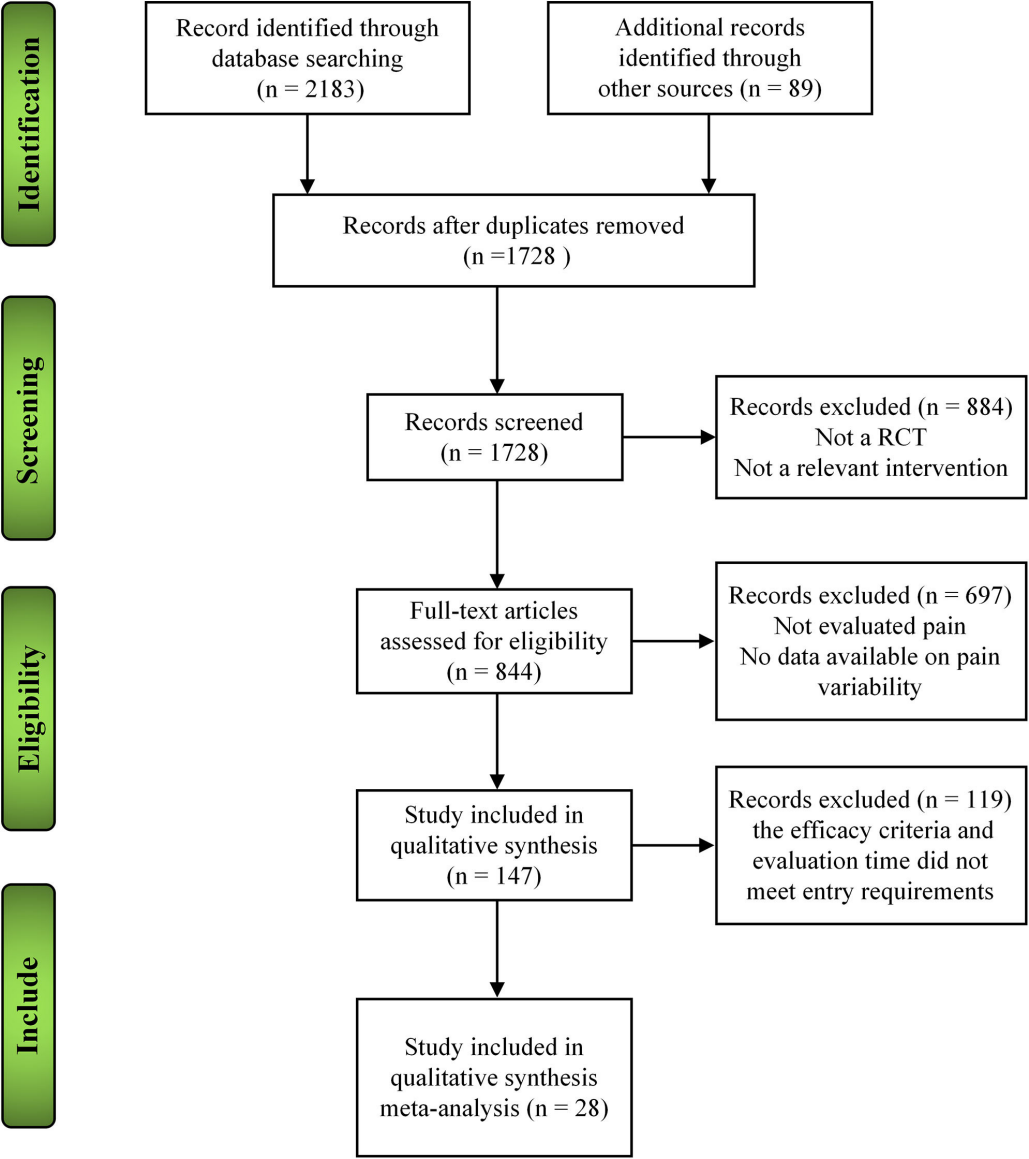
The different phases of the search of the 8 databases and the selection of the studies included in the present analyses.

A total of 2,272 reports were found: all were published in journals; 813 were written in English; and 1,459 articles were written in Chinese, including 1,453 journal articles and 56 academic theses. Of the initially obtained literature, 544 articles were excluded because they were repeated; 884 articles were excluded because they were non-RCTs; 697 articles were excluded because the title and abstract indicate that the treatment group of the article used both acupuncture and medication; since the efficacy criteria and evaluation time of acupuncture treatment did not meet our entry requirements, 119 articles were excluded after further reading. Twenty-eight articles were included in the subsequent analyses (Figure 1).

### Basic Characteristics of Eligible Studies

The 28 RCTs included 2,874 patients: 1,396 received acupuncture treatment, 865 were included as medication controls, and 613 underwent sham acupuncture. Concerning the types of acupuncture administered, 24 applied pure acupuncture; three, electric-acupuncture; and one, acupuncture combined with needling collaterals. Regarding the types of control groups included in the analyzed studies, 13 used sham acupuncture, and 18 administered medication (Figure 1). Five of 28 studies were multi-center controlled trials (18–22), and the rest were single-center controlled trials.

For the diagnosis of migraine, three non-Chinese studies adopted internationally recognized diagnostic criteria (23–25), and the remaining 25 Chinese studies used international or Chinese diagnostic criteria. Chinese diagnostic criteria for migraine without aura are as fellow: A. at least 5 attacks in accordance with B-D characteristics; B. headache attacks (untreated or ineffective) lasting for 4 to 72 h; C. at least 2 headache characteristics as follows 1. Unilateral 2. Throbbing 3. Moderate or severe pain 4. Daily activities (such as walking or climbing stairs) will aggravate headache or avoid such activities when headache occurs; D. at least accompanied by headache With the following 1. Nausea and / or vomiting 2. Photophobia and voice fear; E. cannot be attributed to other diseases. As for the efficacy criteria for migraine, FM was evaluated in 14 studies (18, 20, 21, 24, 26–35). Nine studies evaluated DM (18, 22, 27–31, 35, 36). Five studies (29, 36–39) used TCD to evaluate changes in intracranial blood flow, among which two were measured after completion of treatment and the other three were measured 1 month after treatment, the TCD examination was performed outside migraine attacks. The degree of pain exhibited by the patients was measured with the VAS in 16 studies (19, 20, 25, 26, 29, 31, 33–35, 38–44). A total of 17 studies (18, 20, 21, 23, 27–31, 34–39, 43, 45) evaluated the treatment efficiency by referring to the following criteria: clinically curative, the pain disappeared after the treatment and no recurrence was observed after follow-up; effective, the degree of pain is reduced and the frequency/duration of attacks lessened; ineffective: no significant reduction or aggravation of symptoms was observed after treatment. The total effective rate was defined as the sum of the curative and effective rates. A total of four studies (34, 36, 38, 41) reported adverse reactions in the treatment process. Table 1 shows the main characteristics of the included studies: country of study, sample size of the treatment and control groups, withdrawal amount, treatment method selected for the treatment group, treatment method used for the control group, time course of efficacy evaluation, efficacy evaluation index, and follow-up period.

**Table 1:** Characteristics of the included studies in the meta-analysis.

**Study**	**Country**	**Patients (AC vs. MD vs. SAC)**	**Drop out (AC vs. MD vs. SAC)**	**Acupuncture group**	**Medicine group**	**Sham acupuncture group**	**Timing of TCD**	**Duration of acupuncture group**	**Headache measure**	**Follow-up**
Wu et al. (35)	China	40 vs. 40 vs. 0	0	Acupuncture	Flunarizine	/	/	1 month	FM, DM, VAS, ER	1 month
Xiao et al. (45)	China	30 vs. 30 vs. 30	0	Electro-acupuncture	Diclofenac sodium	Non-acupoint	/	1 month	VAS, ER	1 month
Tastan et al. (25)	Turkey	30 vs. 30 vs. 0	6 vs. 0 vs. 0	Acupuncture	Acetaminophen	/	/	1 month	VAS	3 months
Wang et al. (27)	China	20 vs. 0 vs. 20	1 vs. 0 vs. 1	Acupuncture	/	Non-acupoint	/	1 month	FM, DM, VAS	2 months
Shu et al. (45)	China	60 vs. 60 vs. 0	2 vs. 3 vs. 0	Acupuncture	Flunarizine hydrochloride	/	/	1 month	VAS, ER	2 months
Zhang (29)	China	60 vs. 60 vs. 0	0	Acupuncture	Nimodipine + Oryzanol	/	Before and after treatment	1 month	TCD, VAS, ER	1 month
Chen et al. (22)	China	38 vs. 0 vs. 38	0	Acupuncture	/	Non-acupoint	/	1 month	DM, ER, AE	2 months
Ling et al. (46)	China	83 vs. 0 vs. 80	0	Acupuncture	/	Non-acupoint	/	1 month	FM, VAS	2 months
Wei et al. (27)	China	30 vs. 30 vs. 0	0	Acupuncture & blood-letting	Fenbid	/	/	1 month	FM, DM	1 month
Sun et al. (43)	China	30 vs. 30 vs. 0	0	Acupuncture	Ergotamine	/	/	1 month	VAS, ER	1 month
Cai et al. (42)	China	20 vs. 0 vs. 20	1 vs. 0 vs. 1	Acupuncture	/	Non-acupoint	/	1 month	FM, DM, VAS, ER	1 month
Su et al. (31)	China	35 vs. 32 vs. 0	0	Acupuncture	Flunarizine hydrochloride	/	/	1 month	FM, DM, VAS, ER	1 month
Liu et al. (30)	China	58 vs. 58 vs. 0	0	Electro-acupuncture	Nimodipine + aspirin	/	/	1 month	FM, DM, ER	NA
Zhang et al. (38)	China	33 vs. 34 vs. 0	2 vs. 1 vs. 0	Acupuncture	Flunarizine	/	Within 2 days before treatment and 2 days after treatment	1 month	TCD,VAS, ER,AE	1 month
Qu and Shen (41)	China	31 vs. 31 vs. 0	0	Acupuncture	Flunarizine hydrochloride	/	/	1 month	FM, VAS, AE	1 month
Chen S (34)	China	38 vs. 36 vs. 37	1 vs. 2 vs. 2	Acupuncture	Flunarizine hydrochloride	Non-acupoint	/	1 month	FM, VAS, ER, AE	2 months
Chen et al. (28)	China	70 vs. 0 vs. 70	0	Acupuncture	/	Non-acupoint	/	1 month	FM, VAS	2 months
Zhang et al. (14)	China	37 vs. 38 vs. 38	0 vs. 3 vs. 2	Acupuncture	Flunarizine hydrochloride	Non-acupoint	4 weeks before treatment, 4 weeks after treatment and 8 weeks after treatment	1 month	FM, DM, TCD, VAS, ER	2 months
Yang et al. (21)	China	76 vs. 0 vs. 75	0	Acupuncture	/	Non-acupoint	/	1 month	FM, ER	NA
Cao et al. (20)	China	23 vs. 0 vs. 19	0	Electro-acupuncture	/	Non-acupoint	/	1 month	FM, VAS, ER	6 months
Chen et al. (28)	China	30 vs. 0 vs. 29	0	Acupuncture	/	Non-acupoint	/	1 month	FM, DM, ER	1 month
Foroughipour et al. (14)	Iran	50 vs. 0 vs. 50	NA	Acupuncture	/	Non-acupoint	/	1 month	FM	4 months
Zheng et al. (36)	China	60 vs. 60 vs. 0	1 vs. 2 vs. 0	Acupuncture	Flunarizine hydrochloride	/	Before and after treatment	1 month	TCD, ER, AE	1 month
Liu et al. (37)	China	42 vs. 42 vs. 0	0	Acupuncture	Nimodipine + Ergotamine	/	Before and after treatment	1 month	FM, DM, TCD, ER	1 month
Ying L et al. (26)	China	108 vs. 0 vs. 107	0	Acupuncture	/	Non-acupoint	/	1 month	FM, VAS	2 months
Wang et al. (40)	China	70 vs. 70 vs. 0	9 vs. 11 vs. 0	Acupuncture	Flunarizine hydrochloride	/	/	1 month	FM, VAS	1 month
Zhong et al. (18)	China	114 vs. 104 vs. 0	0	Acupuncture	Flunarizine hydrochloride	/	/	1 month	FM, DM, ER	1 month
Allais et al. (23)	Italy	80 vs. 80 vs. 0	3 vs. 7 vs. 0	Acupuncture	Flunarizine hydrochloride	/	/	2 months	FM, ER	6 months

*AC vs. MD vs. SAC: acupuncture group vs. medication group vs. sham acupuncture group; NA, not reported; FM, frequency of migraine; DM, duration of migraine; TCD, transcranial doppler; VAS, visual analog scale; ER, effective rate; AE, adverse events*.

### Quality Assessment

**Figure 2:**
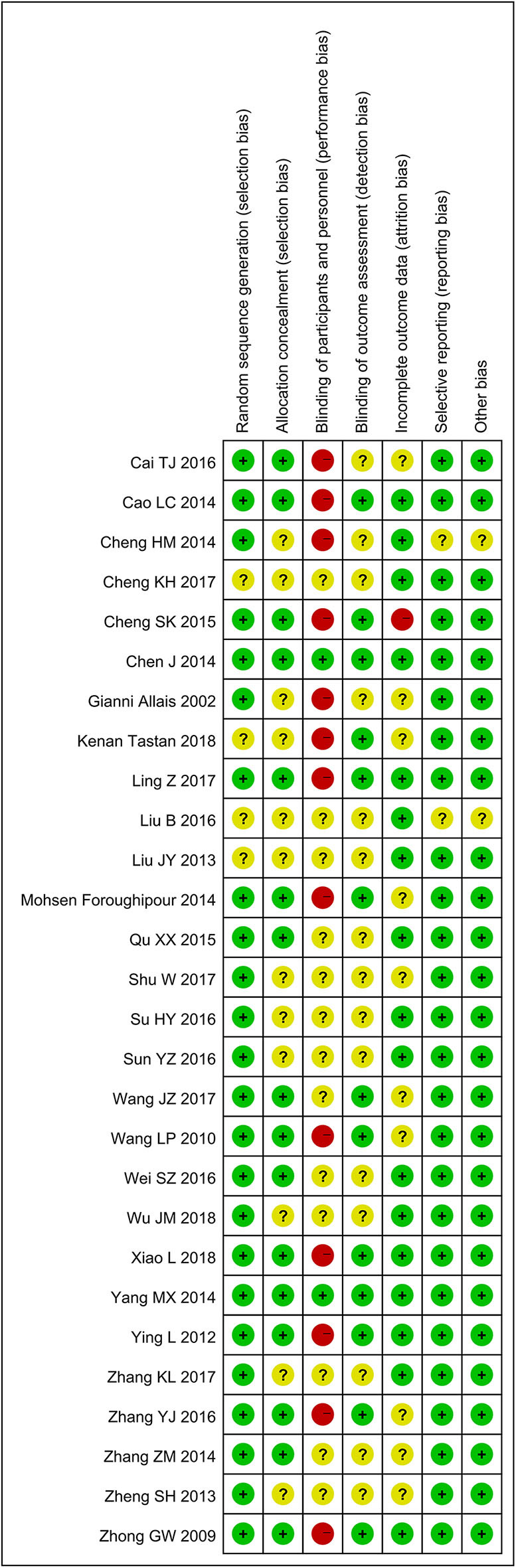
Bias risk summary map.

The results of methodological evaluation are shown in Figure 2. Of the 28 studies, 24 mentioned their methods of random allocation: 10 used a random number table (27, 28, 31, 35, 38, 39, 42–45), 10 used a computer-based method (18, 21, 23, 24, 29, 32, 34, 36, 40, 41), and four used a central random system (19, 20, 26, 33). Thirteen studies were blinded: 11 used a single-blind method (18, 20, 24–26, 32–34, 38, 40, 45); and two, a double-blind method (19, 33). Fifteen articles mentioned their methods of random hiding: 12 used sealed envelopes (18, 24, 26, 27, 29, 32, 34, 38, 40–42, 45); one, a computer-based method (21); and two, a central random system (19, 20). Eleven articles reported the number of people who discontinued the trial (23–25, 29, 32, 34, 36, 38, 40, 42, 44).

### Results of Individual Studies

#### FM

**Figure 3:**
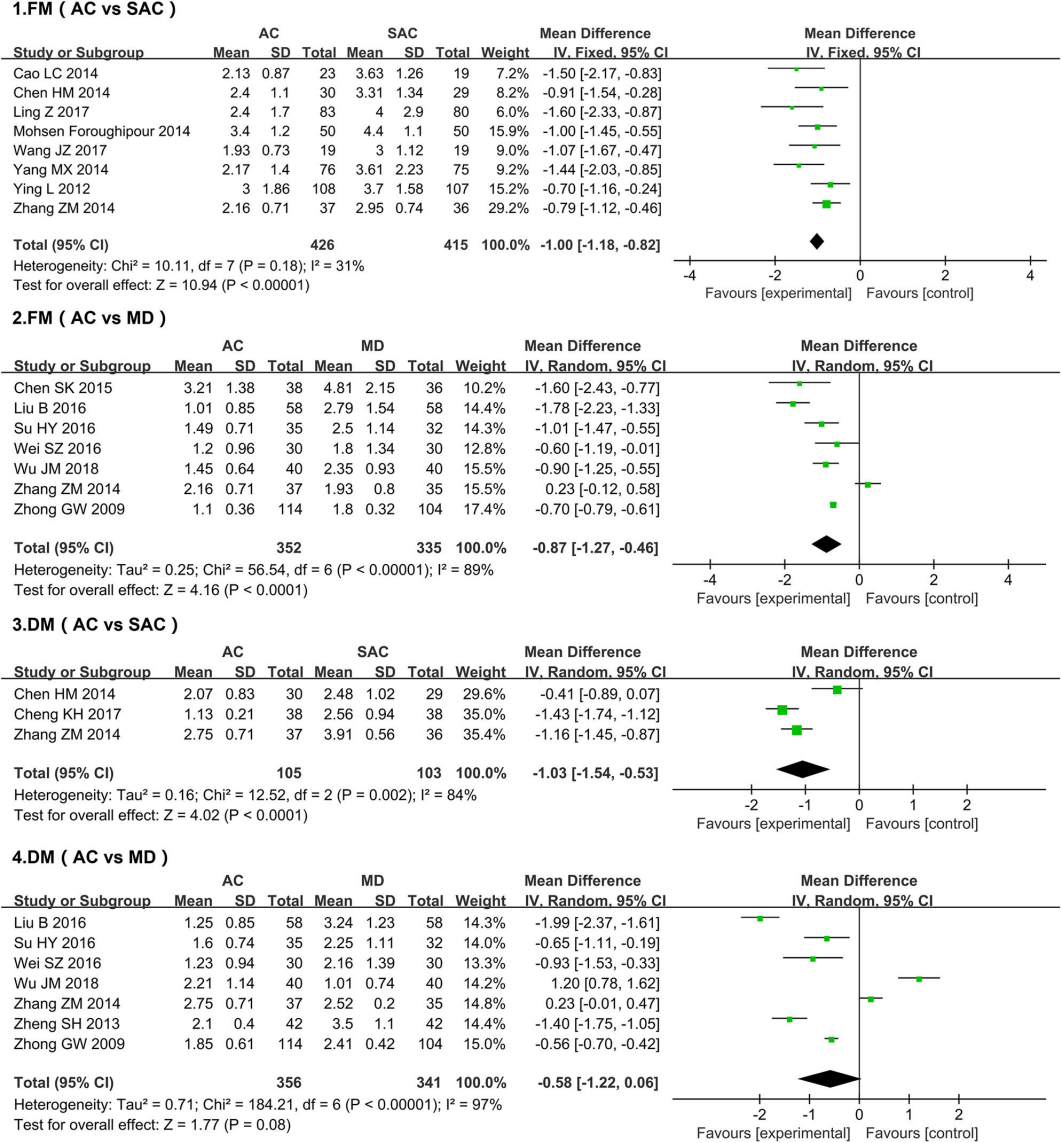
Forest plots of frequency and duration of migraine after acupuncture treatment.

A total of 14 studies (18, 20, 21, 24, 26–35) evaluated FM, comprising a total of 1,528 patients (Figure 3). The acupuncture group was used as the treatment group, and the sham acupuncture group was used as the control group for the chi-square test: χ^2^ = 10.11, I^2^ = 31%, *P* = 0.18 (*P* > 0.05). The forest map generated after statistical combination formed the shape of a diamond situated to the left of the invalid line (MD = −1.00, 95% CI [-1.27,−0.46]), indicating that the improvement of FM in the acupuncture-treatment group was better than that in the sham-acupuncture-treatment group. When comparing the FM of the acupuncture group and the medication group after treatment, the difference between above two groups and their heterogeneity were statistically significant (MD = −0.87l, 95% CI [-1.27,−0.46], *P* < 0.001; I^2^ = 89%, *P* < 0.0001; respectively).

#### DM

Of the studies evaluating the effect of acupuncture on DM (Figure 3), three meta-analyses using sham acupuncture as the control group showed statistical heterogeneity among the results (*P* = 0.002, I^2^ = 84%) (22, 28, 29); therefore a random-effect model was adopted. Results show that, relative to the sham-acupuncture group, acupuncture significantly improved DM; the difference between the two groups was statistically significant (MD = −1.03, 95% CI [-1.54, −0.53], *P* = 0.0009). Although seven studies that administered medication to the control group found that acupuncture did not improve DM more than drug administration (MD = −0.58, 95% CI [-1.22, 0.06], *P* = 0.08 (*P* > 0.05)) (18, 27, 29–31, 35, 36), their results featured significant heterogeneity (I^2^ = 97%, *P* < 0.0001); thus, the relative efficacies of the two methods remains unclear.

#### TCD

**Figure 4:**
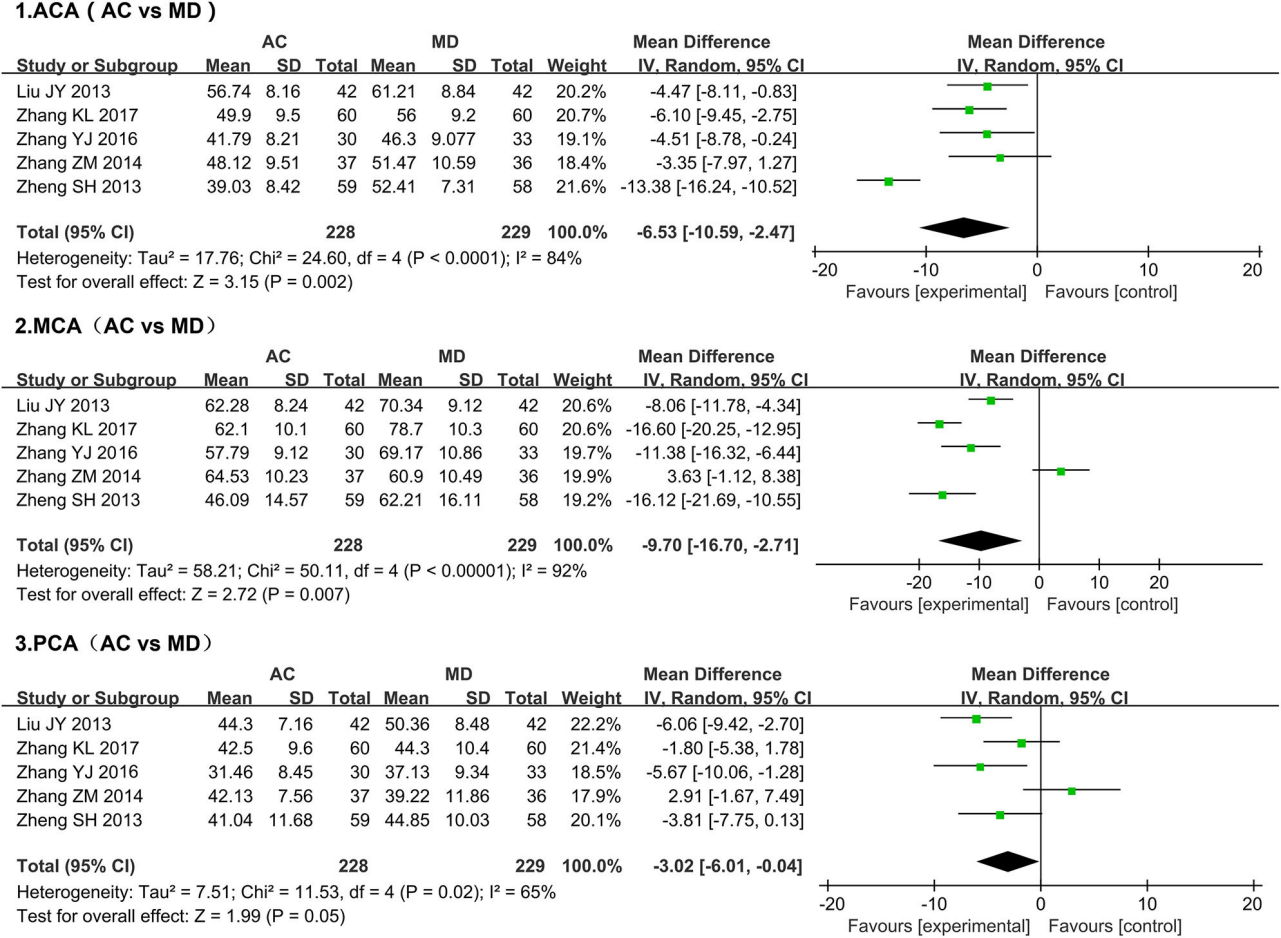
Forest plots of TCD changes after acupuncture treatment.

Given that migraine is characterized by elevated intracranial blood flow velocity and pulsatile headaches, TCD detection of intracranial hemodynamic changes can be used to evaluate the improvement of migraine symptoms (47). We, therefore, employed a meta-analysis to analyze the influence of treatment (acupuncture or medication) on intracranial blood flow changes as measured by TCD (Figure 4). Since sham acupuncture treatment was only administered to controls in one of the studies in which TCD was used, a meta-analysis could not be conducted with such studies. Alternatively, we used the 5 studies whose control groups had been treated with medication (mainly ergotamine and flunarizine hydrochloride) (29, 36–39). All five studies examined blood flow velocity in the bilateral anterior cerebral artery (ACA), middle cerebral artery (MCA), and posterior cerebral artery (PCA) from 0 to 1 month after treatment. The studies collectively included 457 patients. Since the heterogeneity of the three groups of data, as determined by the chi-square test, was found to be significant (ACA, I^2^ = 84%, *P* < 0.0001; MCA, I^2^ = 92%, *P* < 0.00001; PCA, I^2^ = 65%, P = 0.02), the data were analyzed using a random-effects model. Our meta-analysis showed that acupuncture-mediated improvements in intracranial blood flow velocity were superior to those mediated by medication (ACA, MD = −6.53, 95% CI [-10.59,−2.47], *P* = 0.002; MCA, MD = −9.70, 95% CI [-16.70, −2.71], *P* = 0.007; PCA, MD = −3.02, 95% CI [-6.01, −0.04], *P* = 0.05).

#### VAS Score

**Figure 5:**
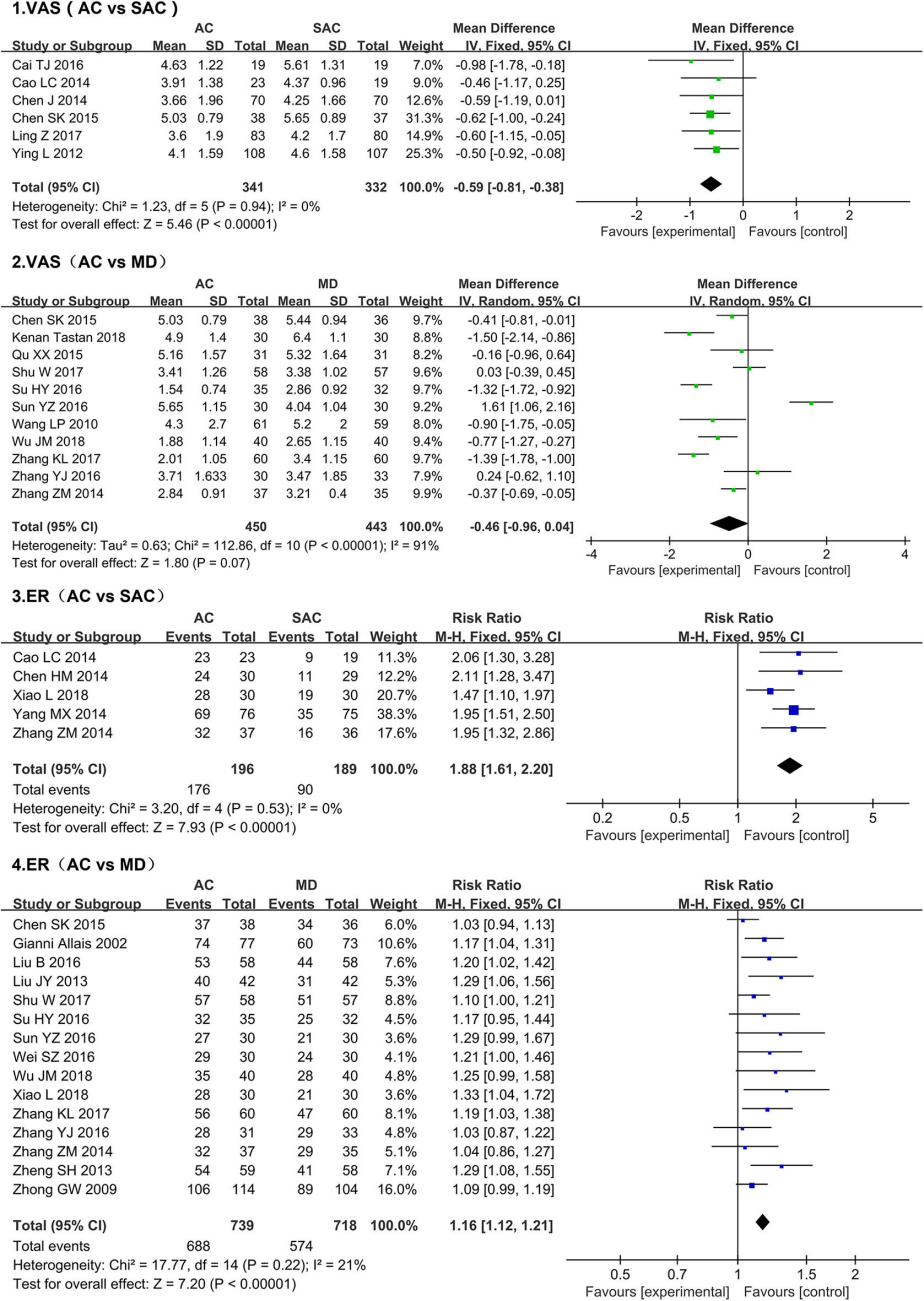
Forest plots of the visual analog scale score and effective rate after treatment.

We conducted a meta-analysis of VAS scores in 16 studies that collectively account for a total of 1,566 people (Figure 5) (19, 20, 25, 26, 29, 31, 33–35, 38–44). The results showed that, compared with the sham acupuncture group, VAS scores in the acupuncture group decreased significantly (MD = −0.59, 95% CI [-0.81, −0.38], *P* < 0.00001); more importantly, no heterogeneity among the studies (I^2^ = 0%, *P* = 0.94). We also conducted a meta-analysis of studies using medication as a treatment method. Due to the large heterogeneity (I^2^ = 91%, *P* < 0.00001), a random-effect model was selected. The results indicated that there was no significant difference in the improvement of VAS score between the medication groups and acupuncture groups (MD = −0.46, 95% CI [-0.96, −0.04], *P* = 0.07).

#### ER

A total of 17 studies of ER (Figure 5) were evaluated: five used sham acupuncture as the control (20, 21, 28, 29, 45), and 15 administered medication to the control group (18, 23, 27, 29–31, 34–39, 43–45). Since the data heterogeneity of the two groups was small (sham acupuncture, I^2^ = 0%, *P* = 0.53; medication, I^2^ = 21%, *P* = 0.22), the fixed-effect model was used for comparisons. Our results showed that, relative to the sham acupuncture and medication, acupuncture achieved a higher rate of effective treatment (sham acupuncture *vs*. acupuncture, MD = 1.88, 95% CI [1.61, 2.20], *P* < 0.00001; medication *vs*. acupuncture, MD = 1.16, 95% CI [1.12, −1.21], *P* < 0.00001).

#### AE

**Figure 6:**
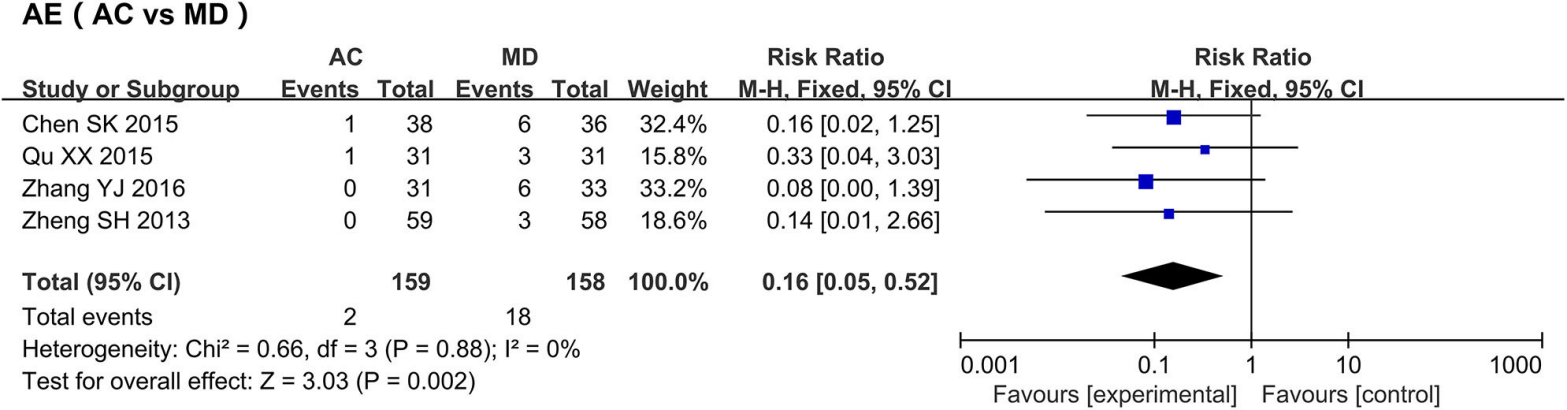
Forest plots of the adverse effects after treatment.

A meta-analysis of the adverse reaction rates in four medication studies was conducted (Figure 6) (34, 36, 38, 41); one study was excluded as it used sham acupuncture as the control group (22). The meta-analysis of the four studies showed that 18 of 158 patients in the medication groups developed adverse reactions (11.39%), while only two of the 159 patients that received acupuncture developed adverse reactions (0.01%); these results indicate that the acupuncture therapy features relatively higher security than medication (RR: 0.16, 95% CI [0.05, −0.52], P = 0.002); the results showed no heterogeneity (I^2^ = 0%, P = 0.88).

### Publishing Bias

We conducted a funnel plot analysis of the literature that administered sham acupuncture and medication to the control groups. The plot was asymmetrical, indicating the existence of publication bias (Supplementary Figure 1).

## Discussion

The results of this meta-analysis suggest that acupuncture treatment is associated with a higher therapeutic efficiency than sham acupuncture. This was demonstrated through the lower frequency of migraine attacks, lower VAS score, and fewer side effects observed in the acupuncture group (compared to the sham acupuncture group). However, although these findings indicate that acupuncture therapy was effective in reducing the duration of headaches, the inter-study heterogeneity was too great to draw a definitive conclusion. When compared with the medication group, the acupuncture group showed higher treatment efficiency and fewer adverse reactions. Further, the acupuncture group also showed greater improvements in headache frequency and VAS scores compared to the medication group. However, the reported reductions in headache duration were similar for the acupuncture and medication groups.

The etiology of migraine is complicated, and there is evidence that its occurrence is related to abnormal cerebral blood shunt, cerebrovascular reactivity and cerebrovascular relaxation stimulation. Beside, the blood flow velocity of the middle cerebral artery is also suggested to be correlated with the development and prognosis of migraine. Therefore, the detection and analysis of intracranial hemodynamics in patients with migraine is the main concern of many researchers (46, 48–51). Given the importance of hemodynamic changes in migraine and the usefulness of their detection for the diagnosis and evaluation of this condition, changes in blood flow velocity detected by TCD were analyzed. Taking the medication group as the control in the five studies included in the TCD meta-analysis, the results showed that acupuncture treatment was associated with better effects with regard to intracranial hemodynamics; ACA, MCA, and PCA blood flow velocity decreased faster after acupuncture than after drug therapy. Although the specific mechanism of the effect of acupuncture on intracranial hemodynamics is not clear, the existing clinical research shows that acupuncture can stimulate brain related functional areas, so as to improve the blood flow after brain injury. In addition, some basic researches show that acupuncture can mediate intracranial oxidative stress, relieve blood vessels and accelerate angiogenesis through a variety of complex molecular pathways (52). The TCD results of this study seem to further support the improvement of intracranial blood flow by acupuncture.

Acupuncture is a broad term that, includes the application of acupuncture needles at several points across the scalp, ear, and abdominal area, as well as Electroacupuncture (53). Moreover, acupuncture has the characteristics of local effect and targeted individual application. Therefore, while the same types of needle were used across studies, different acupoints may have been selected according to the specific therapeutic needs of individual patients. These details were not specifically classified for the included studies. NSAIDs, such as acetaminophen, naproxen or ibuprofen, are recommended for the treatment of mild to moderate migraine. Migraine-specific drugs such as ergot and triptan are recommended for the treatment of moderate to severe headache, allowing for rapid symptomatic improvement (54, 55). Since the condition of most migraine patients is constantly changing, it is difficult to ensure that the medication type and dosage prescribed to each patient included in the present study were consistent throughout the treatment phase. Due to the different treatment habits, some doctors prefer to use a combination of multiple drugs to treat migraine, so as to achieve a faster and more thorough treatment effect, which makes it more difficult to include the research of the same medication treatment methods and acupuncture methods. In addition, during acupuncture treatment, doctors must continuously carry out treatments and give corresponding guidance according to the patients' condition. Therefore, it is extremely difficult to explore the efficacy of acupuncture as a double-blind study, which serves as the main, though inevitable, limitation of this study.

While our results suggest that acupuncture might be more effective than medication in the treatment of migraine, it is important to take into account that there was substantial heterogeneity between the studies analyzed. One of the reasons for this heterogeneity was sample size; although several studies used high quality research methods, most had small sample sizes (~40–60 patients). The fact that there was a lack of multi-center research available to be included in this systematic review adds to this constraint. Therefore, deviation was inevitable in the present analysis. To obtain a comprehensive understanding of the clinical efficacy of acupuncture in the treatment of migraine, future research would benefit from the development of more prospective, multi-center, large-sample RCTs following rigorous study designs. Furthermore, for the effective use of acupuncture as a prophylactic treatment for migraine, it is necessary for the processes of acupoint selection and choice of treatment methods to be standardized based on the Traditional Chinese Medicine theory of syndrome differentiation for treatment. This will improve comparability between studies investigating this type of treatment and allow for quality standards to be more effectively controlled. In addition, efforts are required for the development of a clinical acupuncture treatment program with proven efficacy and high feasibility.

## Conclusions

Overall, the results of our meta-analysis showed that, compared with the sham acupuncture group, the acupuncture group had greater improvements in the frequency of migraine attacks, VAS score, and treatment efficiency. Further, compared with the medication group, the acupuncture group showed higher treatment efficiency and fewer adverse reactions. This study illustrates the efficacy of acupuncture treatment in migraine and the associated hemodynamic changes, providing a theoretical basis for the use of acupuncture as a treatment for migraine.

## Author Contributions

M-QO, W-HF, and F-RS contributed equally to this paper. L-LC and H-HZ setted the research direction and search strategy, F-RS and W-XJ are responsible for the literature search and content screening, M–JL and Y-JC extracted the data from the literature and organized them. S-YL, Y-SY, and M-HL contributed to the analysis of data, quality assessment and bias risk analysis. M-QO, W-HF, and F-RS made the chart and completed the writing of the manuscript. All authors contributed to the article and approved the submitted version.

## Conflict of Interest

The authors declare that the research was conducted in the absence of any commercial or financial relationships that could be construed as a potential conflict of interest.
