# Supplementary material for: A Systematic Review and Meta-analysis of the Therapeutic Effect of Acupuncture on Migraine
Source: Front Neurol. 2020 Jun 30;11:596. doi: 10.3389/fneur.2020.00596 (PMC7344239; doi:10.3389/fneur.2020.00596)
Supplement: Supplementary file 1 [file Table_1.DOCX]

Supplementary Material

# Supplementary Figures and Tables

## Supplementary Figures


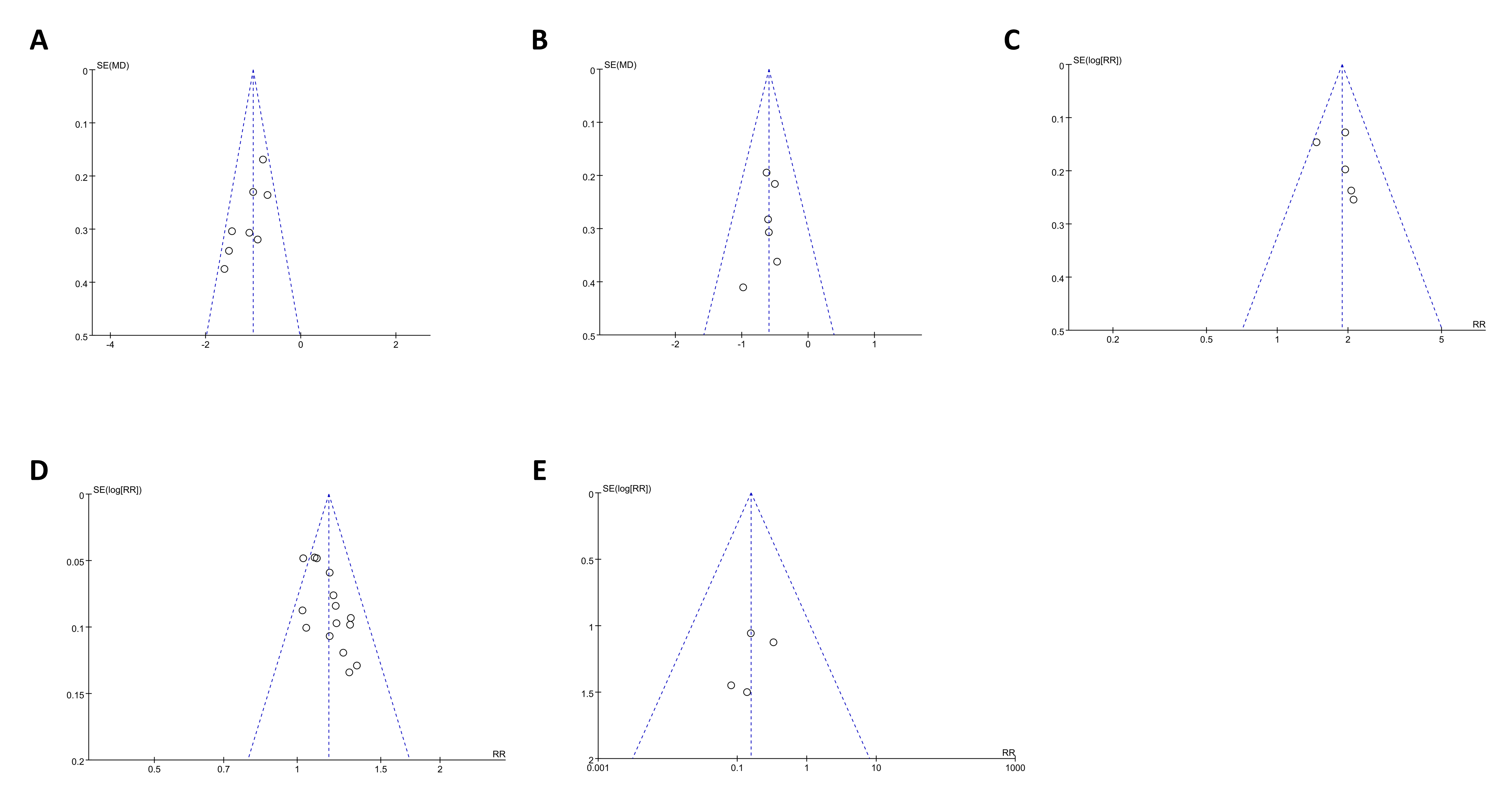


**Supplementary Figure 1.** Publication bias analysis. (A) FM AC VS SAC; (B) VAS AC VS SAC; (C) ER AC VS SAC; (D) ER AC VS MD; (E) AE AC VS MD.
